# Dentists’ Self-Perceived Role in Offering Tobacco Cessation Services: Results From a Nationally Representative Survey, United States, 2010–2011

**DOI:** 10.5888/pcd11.140186

**Published:** 2014-11-06

**Authors:** Deanna P. Jannat-Khah, Jennifer McNeely, Margaret R. Pereyra, Carrigan Parish, Harold A. Pollack, Jamie Ostroff, Lisa Metsch, Donna R. Shelley

**Affiliations:** Author Affiliations: Deanna P. Jannat-Khah, Jennifer McNeely, School of Medicine, Department of Population Health, New York University, New York, New York; Margaret R. Pereyra, Carrigan Parish, Lisa Metsch, Mailman School of Public Health, Department of Sociomedical Sciences, Columbia University, New York, New York; Harold A. Pollack, School of Social Service Administration, University of Chicago, Chicago, Illinois; Jamie Ostroff, Department of Psychiatry and Behavioral Sciences, Memorial Sloan Kettering Cancer Center, New York, New York.

## Abstract

**Introduction:**

Dental visits represent an opportunity to identify and help patients quit smoking, yet dental settings remain an untapped venue for treatment of tobacco dependence. The purpose of this analysis was to assess factors that may influence patterns of tobacco-use–related practice among a national sample of dental providers.

**Methods:**

We surveyed a representative sample of general dentists practicing in the United States (N = 1,802). Multivariable analysis was used to assess correlates of adherence to tobacco use treatment guidelines and to analyze factors that influence providers’ willingness to offer tobacco cessation assistance if reimbursed for this service.

**Results:**

More than 90% of dental providers reported that they routinely ask patients about tobacco use, 76% counsel patients, and 45% routinely offer cessation assistance, defined as referring patients for cessation counseling, providing a cessation prescription, or both. Results from multivariable analysis indicated that cessation assistance was associated with having a practice with 1 or more hygienists, having a chart system that includes a tobacco use question, having received training on treating tobacco dependence, and having positive attitudes toward treating tobacco use. Providers who did not offer assistance but who reported that they would change their practice patterns if sufficiently reimbursed were more likely to be in a group practice, treat patients insured through Medicaid, and have positive attitudes toward treating tobacco dependence.

**Conclusion:**

Findings indicate the potential benefit of increasing training opportunities and promoting system changes to increase involvement of dental providers in conducting tobacco use treatment. Reimbursement models should be tested to assess the effect on dental provider practice patterns.

## Introduction

Dental providers have a credible and central role in providing tobacco cessation services. Most smokers see a dentist annually, and tobacco use is a known risk factor for oral disease (1,2). However, dental care settings remain an untapped venue for the treatment of tobacco dependence (2). Closing this gap in practice is a key objective of *Healthy People 2020*, which for the first time includes goals for improving tobacco screening and cessation counseling rates in dental care settings (3).

The 2008 US Public Health Service Guideline (PHSG), *Treating Tobacco Use and Dependence, *provides strong evidence that delivery of tobacco dependence treatment (TDT) by health care providers — defined as asking all patients about tobacco use, advising smokers to quit, assessing readiness to quit, offering cessation assistance, and arranging follow-up (the 5As) — can produce significant and sustained reductions in tobacco use (4).

Although surveys indicate that dental providers are increasingly adhering to the PHSG by screening for tobacco use, only 10% to 25% routinely deliver cessation assistance (ie, cessation pharmacotherapy prescriptions, referral for counseling, or both) (5,6). In descriptive studies, dentists frequently cite a lack of reimbursement, time constraints, and perceived patient resistance as barriers to adopting cessation treatment guidelines (5–11). In the few studies that analyzed factors that may influence dentists’ adherence to tobacco use treatment guidelines, a lack of training and a lack of confidence in their ability to help patients quit smoking are consistently associated with low rates of cessation intervention delivery (6–9). Most of these studies, however, included small or nonrepresentative samples.

The PHSG strongly recommends system-level changes and provider reimbursement for optimal integration of TDT into routine preventive care services (4). More broadly, medical and dental practices alike are undergoing rapid systems-level reforms, including adoption of “meaningful use” standards and use of financial incentives that change the provision of care (12). However, few studies of tobacco cessation activities performed by dentists focused on identifying practice and policy-level changes that may influence provider behavior (13,14). To fill gaps in this literature, we conducted the largest national representative survey, to date, of primary care dentists to examine correlates of tobacco-use–related practice patterns and willingness to provide cessation assistance if sufficiently reimbursed.

## Methods

### Study design

We surveyed a nationally representative sample of dentists in the United States. The survey examined dentists’ knowledge, attitudes, and beliefs about offering oral rapid HIV testing and other preventive screening behaviors, including TDT (15). This article focuses on the responses to the TDT questions. The institutional review boards of the University of Chicago, the University of Miami, and Columbia University approved this study.

### Participants

We obtained the sampling frame from the American Dental Association (ADA) Survey Center (ADASC). The ADASC provided a stratified random sample of dentists on the basis of 2 variables: urbanicity and practice type. Practice type included private practitioners, who comprise 95% of the ADA sampling frame, and public health practitioners. The public health stratum included 80% of the 383 US dentists who identified themselves as “public health dentists,” defined as dentists who identified as state or local government employees; as being part of a hospital medical group, health care clinic, or public health dentistry; or as safety net providers. Urbanicity included Ryan White CARE Act–eligible metropolitan areas (EMAs) and a mixture of other metropolitan and rural areas. Because the objective of the parent study was to assess willingness to provide oral rapid HIV screening, geographic locations with high HIV/AIDS prevalence were oversampled to capture data on the attitudes and practices of practitioners most likely exposed to HIV-positive patient populations. Therefore, 75% of the sample was drawn from Ryan White CARE Act EMAs.

### Data collection

The National Opinion Research Center at the University of Chicago conducted the survey from November 2010 through November 2011. The survey contacted a sampling frame of 2,876 dentists, using the standard 5 contacts in the order recommended by Dillman (16). These contacts included a prenotification letter, initial survey mailing, thank you or reminder letter, signature-confirmed replacement survey, and final follow-up via telephone. We supplemented follow-up contacts with mass faxes, e-mails and postcards. Respondents were also given the option of completing the survey electronically via a password-protected Internet site. A $10 cash incentive was included in the initial questionnaire mailing and was increased to $20, then $50, and finally $100 with each round of mailings.

### Measures

The survey assessed several provider-level characteristics, including age, sex, professional education, number of hours of TDT-related training, self-rated clinical knowledge, and attitudes toward TDT. Using a 4-item Likert scale, which ranged from strongly agree to strongly disagree, attitude items assessed dentists’ perception of provider and patient subjective norms associated with offering TDT and beliefs about whether TDT should be part of the dental professional role. Scores on individual attitude items were averaged to produce a composite variable for the multivariable analyses. For this variable, we reversed negative questions so that a high summary score indicated more positive attitudes about TDT.

Measures of practice characteristics included the number of dental hygienists in the practice, the number of dentists in the practice, acceptance of patients insured through Medicaid (yes or no), and type of practice setting (solo private, group private, or public health). We also asked if the patient chart included a standard screening question about tobacco use (yes or no).

Dentists’ practice patterns were assessed with 4 questions that asked if they routinely performed the following: ask about tobacco use; counsel smokers to quit, defined as advising patients to quit tobacco; offer a referral for counseling, and offer cessation pharmacotherapy. Offering cessation assistance was defined by a positive response to offering a referral for additional counseling, a prescription for cessation medication, or both. Dentists were also asked if they would be willing to offer cessation assistance (yes or no) if sufficient third-party reimbursement were available.

### Data analysis

Base weights were computed for the sample of dentists selected from the ADASC sampling frame to account for the stratified nature of the sample. Univariate and bivariate associations were evaluated using the Pearson χ^2^ test of independence calculated using Stata version 12 (StataCorp, LP). We examined the bivariate associations between provider and practice characteristics with currently providing tobacco cessation assistance and willingness to offer tobacco cessation services if sufficient third-party reimbursement was offered among those not already offering cessation assistance.

We developed 2 logistic regression models. Model A included the entire sample of dentists surveyed with complete data (n = 1,548); the dependent variable was respondents currently offering tobacco cessation assistance. Model B was restricted to providers with complete data who were not currently offering tobacco cessation assistance (n = 864); the dependent variable was willingness to offer tobacco cessation services if sufficient third-party reimbursement were offered. Multivariable logistic regression analyses accounted for sample weights and stratification using the SVY routines. Marginal effects were calculated, and significance was set at *P* < .05.

## Results

Among the 2,876 dentists contacted, 328 were determined to be ineligible (because of either type of practice or expired or revoked dental license), 11 submitted incomplete surveys, and 735 were nonresponders (explicit refusals, unable to be located, or unavailable). In total, 1,802 (70.7%) dentists completed interviews. Most of the questionnaires were completed via mail (n = 1,349). Additional responses were received via the Internet (n = 381), telephone (n = 28), fax (n = 30), or in person (n = 14). Most respondents were male (78.3%), white (78.6%), and aged 45 to 64 years (62.0%) (Table 1). Most dentists surveyed spent more than 35 hours in patient care per week (42.3%), worked in solo practices (66.4%), and did not see patients insured through Medicaid (71.6%). More than 85% of respondents surveyed reported their knowledge of TDT as moderate to excellent. Most respondents reported having had some TDT training; 30.8% reported 1 to 4 hours and 27.6% report more than 5 hours (Table 1).

**Table 1:** Provider and Practice Characteristics From a National Survey of Dentists, United States, 2010–2011

Variable	Overall		Cessation Assistance^a^			Willingness to Offer Assistance if Reimbursement Is Provided^b^		
	Weighted %	N	Weighted %	*P* Value^c^	N	Weighted %	*P* Value^c^	N
**Provider Characteristics**								
**Age, y**								
25–44	26.6	1,670	49.2	.26	1,657	73.3	<.001	918
45–64	62.0		43.6			48.4		
≥65	11.4		39.5			49.9		
**Sex**								
Male	78.3	1,676	45.8	.23	1,663	51.8	.03	922
Female	21.7		40.6			63.8		
**Race/ethnicity**								
White	78.6	1,657	46.6	.08	1,644	51.5	.01	915
Black	3.5		35.4			81.7		
Asian	13.7		34.3			60.1		
Other^d^	4.2		44.0			66.6		
Hispanic or Latino	4.2	1,661	44.3	.97	1,648	68.3	.19	916
**No. of hours of TDT training**								
<1	41.6	1,672	25.3	<.001	1,651	47.9	.01	916
1–4	30.8		52.5			60.8		
≥5	27.6		64.2			65.7		
**Knowledge of TDT**								
None or limited	14.8	1,678	12.9	<.001	1,658	34.4	<.001	920
Moderate to excellent	85.2		50.3			61.5		
**No. of patients seen in a typical week**								
<35	21.2	1,609	38.1	.03	1,596	50.7	.28	887
35–49	21.9		38.3			52.0		
50–74	26.9		48.5			63.0		
≥75	30.0		50.0			53.0		
**No. of hours spent in direct patient care in a typical week**								
≤30	34.1	1,649	44.5	.99	1,630	45.6	.02	901
31–34	23.6		44.4			56.5		
≥35	42.3		44.8			62.4		
**Provider Attitudes^e^**								
**Tobacco use should be part of the dentist’s role.**								
Agree	77.9	1,669	27.7	<.001	1,649	62.5	<.001	916
Disagree	22.1		49.6			36.9		
**If I were to offer tobacco cessation counseling . . .**								
I would be concerned about negative reactions from my patients.								
Agree	14.6	1,662	18.6	<.001	1,652	35.8	<.001	921
Disagree	85.4		48.9			60.2		
I would be doing something positive for the patient.								
Agree	96.4	1,663	45.4	.16	1,654	56.8	<.001	919
Disagree	3.65		30.7			15.7		
My patients’ perception of me as a health care provider would improve.								
Agree	80.2	1,646	48.4	<.001	1,637	64.5	<.001	910
Disagree	19.8		29.9			29.9		
My colleagues’ perception of me as a health care provider would improve.								
Agree	66.3	1,636	49.6	<.001	1,627	66.5	<.001	902
Disagree	33.7		35.2			38.1		
**Practice Characteristics**								
**No. of dentists in practice**								
1	55.8	1,663	43.3	.61	1,650	50.2	.09	916
2	26.8		44.4			62.3		
≥3	17.4		48.2			58.0		
**No. of hygienists in practice**								
0	22.0	1,448	31.3	<.001	1,437	54.6	.95	804
1	28.8		39.7			56.6		
2	31.8		50.9			55.9		
≥3	17.4		52.1			51.9		
**Primary practice setting**								
Solo private practice	66.4	1,672	43.0	.13	1,659	50.9	.01	920
Group private practice	30.5		45.9			64.3		
Public health practice^f^	3.2		61.8			38.0		
**Patient chart includes tobacco use question**								
Yes	91.7	1,681	47.2	<.001	1,662	55.2	.36	922
No	8.3		16.3			48.7		
**Medicaid patients seen**								
Yes	28.4	1,655	39.9	.10	1,635	64.8	.01	912
No	71.6		46.4			49.7		

Abbreviation: TDT, tobacco dependence treatment.

a Cessation assistance is defined as prescribing pharmacotherapy and/or making a referral to a quitline.

b Included providers who do not currently provide cessation assistance.

c *P* values calculated using Pearson χ^2^ test of independence.

d “Other” is defined as American Indian or Alaska Native, Native Hawaiian or Pacific Islander, and “other.”

e “Agree” comprised all responses of “agree” and “strongly agree,” and “disagree” comprised all responses of “disagree” or “strongly disagree.”

f Defined as a dentist who identified as a state or local government employee, hospital medical group, health care clinic, public health dentistry, or a safety net provider.

Overall, respondents reported positive attitudes and perceptions about providing tobacco cessation counseling: 77.9% agreed that TDT should be part of a dentist’s role, 96.4% agreed that they would be doing something positive for their patient by offering tobacco cessation counseling, 80.2% agreed that their patients’ perception of them would improve; and 66.3% agreed that their colleagues’ perception of them as a health care provider would improve. More than 90% of respondents reported having a chart system that includes a standard screening question about tobacco use (Table 1).

Cessation assistance was associated with the following provider characteristics: positive attitudes toward TDT (*P* < .001), a higher level of self-reported TDT knowledge (*P* < .001), more tobacco-related training hours received (*P* < .001), and larger patient volume (*P* = .03). Practice characteristics associated with cessation assistance included having 1 or more dental hygienists in the practice (*P* < .001) and a dental chart that included a tobacco use question (*P* < .001) (Table 1). Provider and practice characteristics associated with willingness to offer cessation assistance, among only those providers who did not currently provide assistance, were younger age (*P* < .001), female sex (*P* = .03), minority race/ethnicity (*P* = .01), more TDT training hours (*P* = .01), positive attitudes (*P* < .001), more hours spent in direct patient care (*P* = .02), being in a group practice (*P* = .01), and treating patients insured through Medicaid (*P* = .01) (Table 1).

Ninety-two percent of dental providers reported routinely screening for tobacco use, and 45% reported offering cessation assistance; 25% reported prescribing pharmacotherapy, and 32% reported referring patients to a quitline. Among those who were not currently providing assistance, nearly 55% (n = 926), reported they would be willing to change their practice patterns if sufficiently reimbursed (Table 2).

**Table 2:** Self-Reported Tobacco Use Treatment Patterns From a National Survey of Dentists, United States, 2010–2011

Variable	Overall	
	Weighted %	N
Ask about tobacco use	92.2	1,678
Counsel patients to quit	76.8	1,674
Prescribe pharmacotherapy	25.0	1,670
Refer to a quitline^a^	32.1	1,672
Cessation assistance^b^	44.6	1,668
Willing to offer cessation assistance if reimbursed^c^	54.7	926

a A quitline is a state or national telephone number that offers tobacco users free telephonic tobacco cessation counseling.

b Assistance is defined as prescribing pharmacotherapy and/or making a referral to a quitline.

c Included providers who denied currently offering cessation assistance.

In multivariable analyses (Table 3), among all dentists surveyed (Model A), the likelihood of offering smoking cessation assistance was significantly higher among those having a chart system that included a screening question about tobacco use compared with those without such a system (β = 0.199, *P* < .001) and among those with positive attitudes about TDT (β = 0.152, *P* < .001). The probability of providing cessation assistance also significantly increased with increasing hours of TDT-related training. Similarly, providers who indicated that their rating of TDT knowledge was moderate to excellent were more likely (β = 0.225, *P* < .001) to provide assistance than providers who responded none to limited. Among providers who did not report currently offering cessation assistance (Model B), willingness to change this behavior with reimbursement was associated with more positive attitudes toward TDT (β = 0.266, *P* < .001). Higher levels of knowledge (β = 0.127, *P* < .05), working in a private group practice (β = 0.167, *P* < .001), and caring for patients insured through Medicaid (β = 0.147, *P* < .01) were also associated with increased willingness to provide this service if offered reimbursement (Table 3).

**Table 3:** Results From 2 Multivariate Logistic Regression Models From a National Survey Of Dentists, United States, 2010–2011

Respondent Characteristic	Cessation Assistance^a^			Willingness to Offer Assistance if Reimbursement Is Provided^b^		
	Linearized Logit Coefficient	Standard Error	*P* Value^c^	Linearized Logit Coefficient^c^	Standard Error	*P* Value^c^
**Age, y**	0.002	0.002	.13	−0.008	0.002	<.001
**Sex**						
Female	1 [Reference]	—		1 [Reference]	—	
Male	0.044	0.045	.33	0.015	0.061	.80
**Race/ethnicity**						
White	1 [Reference]	—		1 [Reference]	—	
Black	−0.102	0.119	.39	0.191	0.105	.07
Asian	−0.027	0.057	.64	−0.080	0.075	.28
Other^d^	0.009	0.063	.89	−0.068	0.078	.38
**Training on tobacco cessation, hours**						
<1	1 [Reference]	—		1 [Reference]	—	
1–4	0.170	0.039	<.001	−0.039	0.052	.45
≥5	0.239	0.041	<.001	−0.048	0.061	.43
**Knowledge of TDT moderate to excellent**	0.225	0.062	<.001	0.127	0.052	.01
**Has positive attitudes toward TDT**	0.152	0.033	<.001	0.266	0.046	<.001
**Patient chart includes tobacco use question**	0.199	0.073	.006	−0.055	0.063	.38
**No. of hygienists**						
0	1 [Reference]	—		1 [Reference]	—	
1	−0.024	0.049	.62	0.012	0.058	.84
2	0.063	0.049	.19	−0.012	0.063	.85
3	0.079	0.053	.13	−0.128	0.067	.06
**Counsel patients to quit using tobacco**	—			0.140	0.042	.001
Medicaid patients seen (0 = no; 1 = yes)	−0.094	0.040	.02	0.147	0.052	.005
**Primary practice setting**						
Solo private practice	1 [Reference]	—		1 [Reference]	—	
Group private practice	−0.022	0.040	.59	0.167	0.052	.001
Public health practice^e^	0.155	0.080	.05	−0.186	0.092	.04

Abbreviation: TDT, tobacco dependence treatment; —, does not apply.

a Cessation assistance is defined as prescribing pharmacotherapy, making a referral to a quitline, or both.

b Included providers who denied currently offering cessation assistance.

c *P* values calculated using a multiple logistic regression model.

d “Other” is defined as American Indian or Alaska Native, Native Hawaiian or Pacific Islander, and “other.”

e Public health practice is defined as a dentist who identified as a state or local government employee, hospital medical group, health care clinic, public health dentistry, or a safety net provider.

## Discussion

This study is the largest national survey of dentists to capture data on practice patterns and attitudes about TDT and the first nationally representative survey analyzing practice (eg, chart systems) and potential policy-level (ie, reimbursement) influences on providing cessation assistance. Participants reported rates of screening for tobacco use that were higher than previous reports (5), which may be related to the widespread integration of clinical reminder systems in these practices (91.7%). Studies conducted primarily in medical care settings demonstrate strong associations between chart reminder systems and significant increases in routine screening for tobacco use (4). Our findings support the inclusion of dental practices in federal programs such as “meaningful use,” which regulates the inclusion of tobacco screening as a core measure in electronic health records (12).

Study participants also reported rates of tobacco cessation counseling that already surpass the *Healthy People 2020* target of 39.3% (3). It is possible that when dentists reported that they routinely counsel patients they could have been referring to a range of activities, from brief advice to more intensive counseling. The vague wording of this question may have led us to overestimate the level of support offered by dentists. For example, rates of referral to evidence-based programs like state quitlines and the frequency of offering a cessation prescription were below 35%.

Reorganizing the practice environment to help clinicians refer and delegate the time-consuming steps of counseling and arranging follow-up may improve rates of TDT and enhance the support smokers receive. Several studies in medical care delivery settings have shown that this can be accomplished by linking patients with statewide telephone counseling programs (17–20). Telephone quitlines, which offer free telephone counseling and often nicotine replacement therapy, are effective in increasing cessation and are now available in all 50 US states (4). Tobacco quitlines, however, are underused by dental providers and should include dental health care settings in their educational outreach to increase adoption of these evidence-based services.

Although implementing referral systems could enhance treatment, changing practice patterns also requires more attention to deficits in tobacco treatment knowledge among dentists. Several studies, including ours, found associations between low rates of adherence to cessation assistance guidelines and a lack of training (2). To address this persistent barrier to adoption of TDT guidelines, it is important to include tobacco use prevention and treatment in the dental school curriculum and to integrate tobacco cessation as a routine part of care in the clinical settings in which students train (21,22). New York State’s continuing education requirement that dentists take a course on tobacco use treatment to renew their license provides accountability and incentive for practicing dentists to obtain additional training and could be widely implemented to other regions nationally (23).

We found that staffing patterns in dental practices may influence adherence to PHSG. Consistent with findings from a study of practices in a National Dental Practice-Based Research Network (24), we found that larger group practices and those with 1 or more dental hygienists were more likely to offer cessation assistance. This finding may indicate greater capacity to achieve tobacco use treatment goals when dentists can delegate this activity to a hygienist. Dental hygiene visits are often “well” visits, last longer, and focus on preventive behaviors (25). Moreover, dental hygienists may be more likely to adhere to the full spectrum of tobacco use screening and treatment guidelines than do dentists (5,25). This adherence may result from the American Dental Hygienists’ Association’s aggressive promotion of training dental hygienists to link smokers to evidence-based treatment through their National Tobacco Intervention Initiative (Ask, Advise, Refer) (26). This program could be adopted by the ADA as a way of reinforcing dentists’ roles in treating tobacco use while increasing awareness about referral resources.

Another potential strategy for improving the quality of TDT is to offer dentists reimbursement for this preventive service. The consensus report from the 2nd European Workshop on Tobacco Use Prevention and Cessation for Oral Health Professionals included a statement emphasizing the importance of appropriate compensation for TDT among oral health providers to assist their tobacco-using patients (27). Before our survey, only 1 study examined factors associated with changing practice patterns if reimbursement were to become available for providing cessation assistance (11). This study was conducted among dentists who were members of a practice-based research network and is therefore not representative of the general dental provider population. In our study, more than 50% of dentists who were not currently offering cessation assistance reported that they would change their behavior if reimbursement were available.

Although public and private insurance coverage and provider reimbursement of TDT has increased in the past decade, these programs have largely excluded dental professionals and dental patients (28,29)_._ For example, Medicare reimbursement is available only to physicians, physician assistants, nurse practitioners, and clinical nurse specialists. Our study suggests that expansion of coverage to include dental settings could significantly increase provision of tobacco cessation assistance.

Notably, dentists in a practice that accepts Medicaid were more willing than other dentists to offer cessation services if reimbursed. Several programs serving Medicaid patients offer reimbursement to dentists for tobacco cessation counseling services. For example, in Pennsylvania, dentists are reimbursed for up to 70 counseling sessions per year (29). As of April 2014, New York State began reimbursing dentists for providing brief counseling when the activities of ask, assess, assist, and arrange are documented in the patient’s chart (30).

As a policy change, reimbursement may be an effective method to drive dental provider-level changes in TDT. However, our findings point to additional factors that may need to be addressed in parallel to this type of policy change, including training to increase knowledge and change attitudes, both of which were correlated with being willing to change TDT practice if reimbursement were made available. There is a need to study the impact and the effectiveness of these novel preventive care and reimbursement models in dental settings.

This study had several limitations. First, providers who use tobacco themselves are less likely to adhere to tobacco use treatment guidelines; however, we did not ask this question and therefore were unable to analyze its impact (5). Second, responses could not be validated and may have been subject to recall and social desirability bias. Third, we asked about willingness to provide tobacco assistance if sufficient reimbursement were available but did not attempt to measure what level of reimbursement would be required to change provider behavior. Examining varying levels of reimbursement may be a determinant of future policy and research. Finally, our focus on locations with a high prevalence of HIV/AIDS may limit generalizability of the findings. These limitations should be balanced against the study’s considerable methodological strengths and clinical importance, including the large nationally representative sample, high rate of provider participation, and attention to the often-cited barrier of reimbursement for delivery of tobacco treatment.

Dental visits are historically underused opportunities to address patients’ tobacco use. *Healthy People 2020*’s new standard should provide the impetus for additional research to study implementation and dissemination strategies in dental settings and to ensure sustainability of practice improvements.
